# Obituary

**Published:** 1879-07

**Authors:** 


### OBITUARY.

At his residence, Xenia, Ohio, Saturday morning, June 21st, 1879, Samuel Martin, M. D., in the eighty-third year of his age.

Dr. Martin was no ordinary man, and his life in this community has been no ordinary career. In the prime of his manhood, he came among us in 1834, and was immediately recognized as a leading physician by the extensive practice accorded him. He gave his whole mind to the practice of his profession, enlisting into his service an excellent library and the leading medical journals of the day. His patrons soon recognized him as not only their physician, but their friend.

The present generation have no conception of the hardships of practice at that early day. The average road of that day would be regarded as impassable now. A strong horse with a saddle were requisite. Day and night, through mud and storm, did Dr. Martin plod his weary way, till his form became familiar to all; and he continued to be "the man on horseback" till laid aside five years ago by paralysis.

As a teacher of young men, Dr. Martin was earnest, industrious and faithful. At least some of his pupils can recall horse back recitations of twenty to forty miles in length, interrupted only by occasional stops to examine and prescribe for patients. Seven of his pupils attended the funeral, four of them (residents of Xenia) being pall-bearers.

Dr. Martin was always zealous for the honor of the profession, and earnest in promoting its progress. He took an active part even in advancing the various specialities in medicine, and was an active member of the Board of Trustees that organized the Ohio College of Dental Surgery, in Cincinnati.

In Dr. Martin was found the happy combination of a sound and active mind in a healthy body. He was seldom unfit for active duty till assailed by the fatal messenger, some five years ago. In the latter years of his life he manifested patience amid pain, and awaited his deliverance with the hope of the Christain. A gentleman, a scholar, a Christian—he is laid to rest; and a small man can not fill his place. G. W.

